# Validity, reliability, and feasibility of the digital motor performance test (DigiMot)

**DOI:** 10.3389/fspor.2025.1688017

**Published:** 2025-10-28

**Authors:** Thorsten Klein, Max Pauli, Joann Greiner, Anke Hanssen-Doose

**Affiliations:** Institute of Movement and Sport, Karlsruhe University of Education, Karlsruhe, Germany

**Keywords:** physical fitness, videoconference, youth, monitoring, diagnosis

## Abstract

**Introduction:**

Assessing and monitoring physical fitness in children and adolescents requires valid, reliable, and feasible tests. The digital motor performance test (DigiMot) was developed for remote assessment via videoconference. This study evaluated its validity, reliability, and feasibility in children and adolescents.

**Methods:**

A total of 1,751 participants aged 5–17 years took part in this study. They completed four fitness tasks assessing coordination (Jumping Sideways), muscular endurance (Push-ups and Sit-ups), and flexibility (Stand-and-Reach). Validity was assessed by comparing remote and face-to-face performances (*N* = 60). Test–retest reliability was evaluated across two remote sessions (*N* = 96), and interrater reliability was analyzed using video-recorded sessions (*N* = 381). Feasibility was examined based on all available remote assessments (*N* = 1,751).

**Results:**

Concurrent validity was good for Jumping Sideways (ICC = .844), Push-ups (ICC = .718), and Sit-ups (ICC = .831), with almost perfect agreement for Stand-and-Reach (*κ* = .850). Test–retest reliability ranged from good (Push-ups, ICC = .781) to excellent (Sit-ups, ICC = .940; Jumping Sideways, ICC = .913), with almost perfect agreement for Stand-and-Reach (*κ* = .845). Interrater reliability was excellent for Jumping Sideways (ICC = .972) and Push-ups (ICC = .967), good for Sit-ups (ICC = .873), and substantial to almost perfect for Stand-and-Reach (*κ* = .805). Completion rates exceeded 98%, with minimal technical issues.

**Discussion:**

The DigiMot test demonstrates good to excellent validity, reliability, and feasibility, while addressing a relevant gap in remote physical fitness testing for children and adolescents.

## 1 Introduction

The assessment and monitoring of physical fitness in children and adolescents demands scientifically established test batteries or profiles. Prominent international examples include the EUROFIT (1) and the ALPHA test battery (2). Both instruments have been implemented for many years in multiple countries to evaluate the physical fitness of children and adolescents (3, 4). By combining data from millions of participants, European reference percentiles have been established for the EUROFIT (3) and the ALPHA test battery (4). In Germany, a national framework for fitness testing is provided by the MoMo physical fitness test profile (5, 6), for which reference percentiles are also available (7). Despite their recognized benefits, these established test batteries and profiles depend on face-to-face administration, suitable sufficiently large test rooms and specially manufactured test material. This increased demand for resources provides an explanation for why the assessment of physical fitness, despite its relevance for the development and health of adolescents, has not been integrated into numerous large-scale studies (8). During the COVID-19 pandemic, such assessments could not be conducted due to contact restrictions (9). Consequently, the remote assessment of physical fitness has gained increasing attention in research (10), with most studies focusing on adults in telehealth and telerehabilitation contexts (10). The increasing interest in such methods highlights their potential not only as substitutes in constrained situations but also as approaches that facilitate broader participation and accessibility. Beyond temporary restrictions, remote testing can enable participation of children in rural or underserved regions, support large-scale assessments with fewer logistical demands, and improve access for populations with limited opportunities for in-person testing (11, 12).Nevertheless, the repertoire of validated, reliable, and feasible remote physical fitness tests for children and adolescents remains limited. The present study therefore aims to evaluate the validity, reliability, and feasibility of DigiMot, a novel digital motor performance test for remote assessment via videoconference. DigiMot provides a new solution to existing gaps in remote fitness testing and is designed to enable scalable evaluation of physical fitness in children and adolescents.

## 2 Material and methods

### 2.1 Participants

Participants for this study were recruited from multiple sources. Two test–retest studies were conducted: the first in 2021, assessing remote reliability, and the second in 2022, comparing remote and face-to-face assessments. In both cases, participants were recruited from schools (primary and community) near the Karlsruhe University of Education. The participants and their parents/legal guardians were informed about the studies and data protection prior to any assessment, and asked to give their written informed consent to participate in the study. All participants that provided informed consent were eligible to take part in the studies.

To augment the sample sizes, additional participants were drawn from the DigiMot study, a sub-study of the COMO study (13), which remotely assessed the physical fitness of children and adolescents (aged 7–17 years) in Germany between November 2023 and January 2025. All participants of the COMO study and their parents/legal guardians were informed about the COMO study and all sub-studies prior to participation and were asked to provide their written informed consent. Before participation in the DigiMot sub-study, they were again informed about the sub-study and data protection and were asked to provide written informed consent once more.

### 2.2 Digital motor performance test DigiMot

The digital motor performance test DigiMot (14), developed during the COVID-19 pandemic and adapted from the MoMo physical fitness test profile (5, 6). It comprises a set of four physical fitness tasks designed to remotely assess coordination, muscular endurance, and flexibility in children and adolescents. The fitness tasks included were:

(1) Jumping Sideways—number of jumps between two marked areas within 15 s, averaged over two valid attempts.
(2) Push-ups—number of completed push-ups within a 40 s period during a single attempt.
(3) Sit-ups—number of completed sit-ups within a 40 s period during a single attempt.
(4) Stand-and-Reach—assessed based on whether participants could reach ground level with their fingers in two attempts.

All four fitness tasks had previously been validated in face-to-face settings as part of the MoMo study (15). For adaptation to remote administration via videoconference, task selection prioritized safe implementation in the home environment. Additional criteria included compatibility with common household conditions, such as available space and technical infrastructure. The only special equipment required was a non-slip, foldable test mat [183 cm × 61 cm × 0.4 cm; specifically manufactured for this purpose (Yogistar, Wiggensbach, Germany)], which was sent to participants in advance.

**Figure 1:**
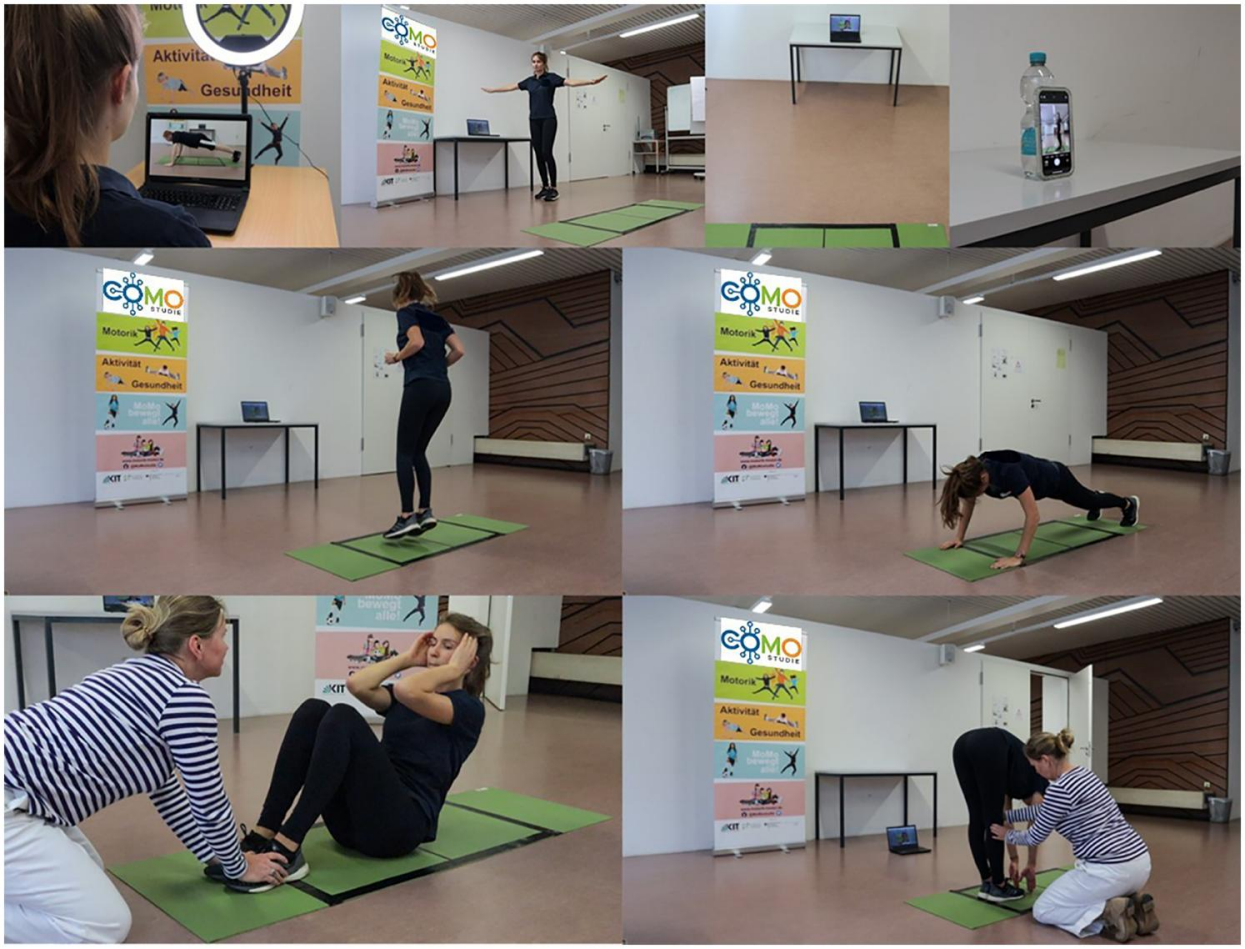
Test setup and implementation of the digital motor performance test DigiMot (10).

At the start of each remote assessment, participants parents/legal guardinas were asked by the test administrator for permission to video-record the assessment for verification of uncertain results (e.g., due to technical issues) and for the evaluation of interrater reliability. Subsequently, participants reported their date of birth, body mass, and height, followed by questions regarding general health status to confirm eligibility. Each fitness task was then explained using standardized instructional videos and demonstrated live by the test administrator. Participants performed a few practice attempts, except for the Stand-and-Reach task, to ensure correct execution. The tests were then administered, and performance was recorded. The complete test setup and implementation of the DigiMot protocol (Figure 1) are described in detail in the DigiMot test manual (4).

### 2.3 Measurement properties

To evaluate the concurrent validity of the DigiMot test, each participant completed both a remote and an in-person assessment. Remote assessments were conducted via Zoom, while in-person assessments took place in the participant's local sports hall. The order of the assessments was not fixed but was individually arranged between the test administrator and the participant. The time interval between the two assessments ranged from a few hours to one month.

Test-retest reliability was assessed through two remote assessments via Zoom, completed by the same participants. The interval between these assessments ranged from 7 to 18 days. Interrater reliability was evaluated by having a second test administrator reassess video recordings of the assessment. During reassessment, the administrator could slow down or pause the videos as needed. Reassessments used to correct test scores in the main dataset were excluded from the reliability analysis.

Feasibility was evaluated by examining the number of remote assessments completed across all data from the DigiMot study and the two test-retest studies.

### 2.4 Statistical analysis

For the Jumping Sideways, Push-ups, and Sit-ups test scores, normality was assessed using Kolmogorov–Smirnov tests, as well as by inspecting skewness, kurtosis, and Q–Q plots. Despite some deviations from normality, concurrent validity, test–retest reliability, and interrater reliability were calculated using two-way mixed-effects intraclass correlation coefficients (ICC) with absolute agreement. This statistical approach is based on previous studies that examined the validity and reliability of remote fitness assessments (16–18) The described method is considered robust against violations of normality in moderate to large samples (19, 20) and aligns with common practices in measurement property research. For interpreting ICCs, we followed the guidelines proposed by Koo and Li (21). In addition to ICCs, Bland–Altman plots were used to visually assess agreement and detect any potential systematic bias between the assessments (21). Furthermore, for all measurement properties, paired *t*-tests were conducted to examine potential learning effects between assessments. For the Stand-and-Reach test, Cohen's Kappa was calculated to assess concurrent validity, test–retest reliability, and interrater reliability. The interpretation of Cohen's Kappa followed the guidelines by Landis and Koch (22). Group comparisons for this categorical outcome were conducted using McNemar's test. All statistical analyses were performed using SPSS (IBM, Version 30), with significance levels set at *p* ≤ 0.05. A *post-hoc* power analysis using G*power (*α* = 0.05), based on the respective results of the correlation analyses, was conducted to confirm that all analyses had sufficient statistical power.

## 3 Results

### 3.1 Sample characteristics

The validity analyses included 60 participants (31 male and 29 female) with a mean age of 10.8 years (±2.6). Of these, 48 participants were from the 2022 test-retest study and 12 from the DigiMot sub-study. The test-retest reliability analyses included a total of 96 participants, of whom 30 were from the 2021 test-retest study and 66 from the DigiMot sub-study. Regarding sex, 43 participants were male and 53 female. The mean age of participants was 11.4 years (±3.3). For the interrater reliability analyses, only participants from the DigiMot sub-study were included (*N* = 381), with a mean age of 10.9 years (±2.8). Regarding sex distribution, 170 were male, 210 female, and one identified as diverse. The feasibility analyses had the largest sample size, with 1,752 participants (78 from the test-retest studies and 1,673 from the DigiMot sub-study). Of these, 826 were male, 927 female, and 2 diverse. Their mean age was 11.2 years (±2.9). All study-specific sample characteristics for the different analyses are presented in Table 1.

**Table 1:** Characteristics of the specific study samples.

Variables	Validity	Test-retest reliability	Interrater reliability	Feasibility
Sample size [*N*]	60	96	381	1,751
Adherence [*N* & %]	60 (96.7)	96 (94.1)	Not applicable	Not applicable
Dropout rate [*N* & %]	2 (3.3)	6 (5.9)	Not applicable	Not applicable
Sex (Male/Female/Diverse) [%]	51.7/48.3/0.0	44.8/55.2/0.0	44.6/55.1/0.3	47.2/52.7/0.1
Age (Mean ± SD) [Years]	10.8 (2.6)	11.4 (3.3)	10.9 (2.8)	11.2 (2.9)
Age groups [*N*]				
5 years	0	1	0	1
6 years	0	5	1	6
7 years	0	8	42	155
8 years	1	7	52	203
9 years	23	8	52	238
10 years	20	13	40	215
11 years	2	9	37	196
12 years	1	10	52	180
13 years	2	6	32	140
14 years	2	8	21	123
15 years	4	7	19	122
16 years	0	9	22	102
17 years	5	5	11	70

### 3.2 Validity

Concurrent validity results for the DigiMot physical fitness tasks are presented in Table 2. Good agreement was found for Jumping Sideways (ICC = .844; *p* < .001), Push-ups (ICC = .718; *p* < .001), and Sit-ups (ICC = .831; *p* < .001). For the Stand-and-Reach, an almost perfect agreement was observed (*κ* = .850; *p* < .001). Group comparisons revealed no statistically significant differences between remote and in-person assessments for any of the physical fitness tasks. The Bland-Altmann plots for Jumping Sideways, Push-ups, and Sit-ups showed also good agreement, no systematic bias, and only a few outliers between the two assessment methods (Supplementary Figures S1–S3).

**Table 2:** Validity of the DigiMot fitness tasks.

Fitness task (assessment condition)	Normality analysis				Correlation analysis				Group comparisons	
	*N*	*P*	Kurtosis	Skewness	*N*	ICC [95%-CI]	Cohen’s kappa	*p*	*N*	*p*
Jumping Sideways (Remote)	59	.200^a^	−.439	.140	59	.844 [.738–.907]	–	<.001	59	.069
Jumping Sideways (Face-to-Face)	59	.071	−.158	.293						
Push-ups (Remote)	59	.060	−.880	.251	59	.718 [.524–.832]	–	<.001	59	.335
Push-ups (Face-to-Face)	59	.200^a^	−.260	.288						
Sit-ups (Remote)	60	.076	1.080	−.967	60	.831 [.717–.899]	–	<.001	60	.207
Sit-ups (Face-to-Face)	60	.013	1.426	−.722						
Stand-and-reach (Remote)	–	–	–	–	60	–	.850	<.001	60	.625
Stand-and-reach (Face-to-Face)	–	–	–	–						

^a^ Lower limit of true significance.

### 3.3 Reliability

Test–retest reliability results are presented in Table 3. Excellent agreement was found for Jumping Sideways (ICC = .913; *p* < .001) and Sit-ups (ICC = .940; *p* < .001), while good agreement was observed for Push-ups (ICC = .781; *p* < .001). The Stand-and-Reach task again showed almost perfect agreement (*κ* = .845; *p* < .001). Group comparisons revealed statistically significant differences between the two assessments for Jumping Sideways, Push-ups, and Sit-ups, but not for Stand-and-Reach. The Bland-Altmann plots for Jumping Sideways, Push-ups, and Sit-ups also showed good agreement, but a bias (higher values) for the second remote assessment (Supplementary Figures S4–S6). Furthermore, no systematic bias and only a few outliers between the two assessment methods were displayed by the Bland-Altmann plots.

**Table 3:** Test-retest reliability of the DigiMot fitness tasks.

Fitness task (assessment condition)	Normality analysis				Correlation analysis				Group comparisons	
	*N*	*p*	Kurtosis	Skewness	*N*	ICC [95%-CI]	Coheńs Kappa	*p*	*N*	*p*
Jumping Sideways (remote T1)	95	.133	.758	−.532	95	.913 [.774–.957]	–	<.001	95	<.001
Jumping Sideways (remote T2)	95	.003	.103	−.744						
Push-ups (remote T1)	96	.025	.794	−.220	96	.781 [.272–.905]	–	<.001	96	<.001
Push-ups (remote T2)	96	.005	1.716	−.682						
Sit-ups (remote T1)	96	<.001	.247	−.599	96	.940 [.874–.967]	–	<.001	96	<.001
Sit-ups (remote T2)	96	.014	.367	−.743						
Stand-and-reach (remote T1)	–	–	–	–	96	–	0.845	<.001	96	.453
Stand-and-reach (remote T2)	–	–	–	–						

Interrater reliability results are shown in Table 4. Excellent agreement was observed for Jumping Sideways (ICC = .972; *p* < .001) and Push-ups (ICC = .967; *p* < .001), and good agreement for Sit-ups (ICC = .873; *p* < .001). For the Stand-and-Reach task, a substantial to almost perfect agreement was found (*κ* = .805; *p* < .001). Group comparisons showed statistically significant differences between raters for Jumping Sideways and Sit-ups, but not for Push-ups or Stand-and-Reach. The Bland-Altmann plots for Jumping Sideways, Push-ups, and Sit-ups showed also good agreement and no systematic bias (Supplementary Figures S7–S9). For the Sit-ups most outliers were above the upper limits of agreement indicating a bias (higher values) for the remote assessment.

**Table 4:** Interrater reliability of the DigiMot fitness tasks.

Fitness task (assessment condition)	Normality analysis				Correlation analysis				Group comparisons	
	*N*	*p*	Kurtosis	Skewness	*N*	ICC [95%-CI]	Cohen’s kappa	*p*	*N*	*p*
Jumping Sideways (remote)	381	.071	.564	−.217	373	.972 [.966–.977]	–	<.001	373	.014
Jumping Sideways (video-recording)	373	.038	.365	−.221						
Push-ups (remote)	381	<.001	.197	.360	381	.967 [.960–.973]	–	<.001	381	.218
Push-ups (video-recording)	381	<.001	−.024	.292						
Sit-ups (remote)	381	<.001	.313	−.255	377	.873 [.841–.899]	–	<.001	377	<.001
Sit-ups (video-recording)	377	<.001	.158	−.444						
Stand-and-reach (remote)	–	–	–	–	380	–	.805	<.001	380	.296
Stand-and-reach (video-recording)	–	–	–	–						

### 3.4 Feasibility

In total, 1,751 remote physical fitness assessments were conducted. Of these, four were aborted due to technical problems or participant withdrawal. Additionally, in 15 assessments, not all fitness tasks were completed for various reasons. Overall, 99.77% of all assessments were completed, and 98.92% were completed without any missing data. Despite minor technical issues, no safety concerns arose during the assessments. Participation was also safely possible for children and adolescents with chronic conditions and overweight.

## 4 Discussion

The present study aimed to evaluate the validity, reliability, and feasibility of the DigiMot test for remotely assessing physical fitness in children and adolescents. Overall, the results demonstrate that DigiMot provides a valid and reliable tool across all included fitness tasks, with feasibility rates exceeding 98%. Comparing our results with previous studies shows that, to our knowledge, the Jumping Sideways test has not previously been evaluated remotely via videoconference, making our study the first to provide validity, reliability, and feasibility metrics for this test. For the Push-up test, our findings align with earlier reports of high validity correlations, reliability coefficients, and completion rates (18, 23, 24). However, methodological variations in test execution (e.g., hand placement, pace, or range of motion) across studies likely account for some differences in absolute scores and completion rates. For the Sit-up test, only one prior study reported comparable feasibility (24), albeit using a longer one-minute version, suggesting that test duration may influence participant performance and completion. In the Stand-and-Reach test, our reliability results were consistent with previous literature (25), indicating robustness across slightly differing administration protocols. These comparisons highlight that methodological standardization is crucial when interpreting remote assessment results and drawing parallels with prior studies.

Overall, our findings support that the DigiMot test can be a robust alternative to in-person testing in children and adolescents. This addresses a notable research gap, which emphasized the lack of high-quality studies focusing on children and adolescents in the context of remote fitness assessments (10). While the review found strong evidence for the validity and reliability of remote assessments in adults, particularly for muscular strength and endurance, only a minority of studies involved participants under 18 years of age (10). Our study contributes critical new evidence to this underrepresented age group, showing that remote methods are not only applicable but also psychometrically sound in school-aged populations. Furthermore, the DigiMot test includes the Stand-and-Reach test to assess flexibility, a fitness component that remains insufficiently evaluated in remote contexts (10) and therefore broadening the spectrum of fitness domains that can be assessed remotely.

### 4.1 Practical implications

The DigiMot test enables remote assessment of physical fitness using videoconferencing tools such as Zoom, requiring minimal equipment and no specialized facilities. A detailed test manual in several languages is available to ensure a standardized and objective test procedure (14). The remote approach of the DigiMot test can enable large-scale monitoring efforts, especially in rural or underserved areas with limited access to sport infrastructure since it reduces logistical barriers and increases accessibility (11, 12). This is reinforced by the high completion rates in our studies and the broader literature (10). Furthermore, our results demonstrate that videorecording and playback functionalities allow for high interrater agreement, flexible scoring, and enhanced examiner training, which may reduce measurement error. Similar results for the interrater reliability were found for a sample of older adults in a telehealth setting (26). The possibility of videorecording the assessment offers a flexible, asynchronous solution to minimize scoring inconsistencies, enhance data quality, and supports remote examiner training. Finally, the overall good results for the measurement properties of the DigiMot test in children and adolescents suggest that it could potentially serve as a scalable tool for physical fitness surveillance programs, health screening, or even as a component of telehealth services in pediatric populations. Our findings underscore the practical advantages of remote testing, while also highlighting methodological constraints—such as reliance on stable internet connections—which must be considered when implementing remote assessments in diverse populations.

### 4.2 Limitations

Despite the promising findings, several limitations must be acknowledged. First, the sample size for the validity analyses was relatively small compared to the sample sizes of our reliability and feasibility analyses. Additionally, the sample distributions among the different ages is uneven (mostly between the ages 8 and 12 years). This may affect the generalizability of some findings and warrants replication in larger and more diverse samples. Second, significant differences in repeated measures for the test-retest reliability for all fitness tasks, except the Stand-and-Reach, suggest the potential for learning or motivation effects. Implementing standardized motivational scripts and more uniform time intervals between the two assessments could help mitigate this issue. Regarding the results of the validity analyses, it must be taken into account that the time interval between the two assessments varied considerably (from a few hours up to one month). Although most assessments were completed within 7–14 days, such variability may introduce biases due to learning effects. Therefore, the time interval between assessments should be more standardized in future research. Additionally, the significant differences in repeated measures for the interrater reliability for the Jumping Sideways and Sit-ups may be due to the use of slow motion and pausing the video recordings, which allowed performances to be recorded more accurately and therefore explaining the significant differences. In general, the remote setting of the DigiMot test may introduce potential biases in the collected fitness data. As each test depends on the participants' technical equipment, varying camera angles, field of view, or poor internet quality can result in incorrect execution of the fitness tasks or hinder accurate counting of repetitions (particularly in Jumping Sideways). Finally, although rare, technical issues and incomplete assessments were observed. While these did not affect overall safety or feasibility rates, they highlight the importance of digital access and literacy.

## 5 Conclusion

The DigiMot test demonstrates promising validity, reliability, and feasibility for the remote assessment of physical fitness in children and adolescents. Across all tested tasks, agreement levels were good to excellent, and completion rates exceeded 98%. These results provide evidence that the DigiMot test can serve as a viable alternative to traditional in-person testing, particularly in contexts where logistical, geographical, or health-related barriers limit face-to-face contact. By offering a standardized, easily accessible, and scalable approach, the DigiMot test has the potential to contribute to large-scale fitness monitoring, health screening, and telehealth applications in children and adolescents. Nevertheless, we do not regard remote fitness tests as a replacement for established in-person assessments. Rather, given their low resource requirements and broad applicability, we see them as a complementary addition that deserves a permanent place in the repertoire of physical fitness testing.

## Data Availability

The raw data supporting the conclusions of this article will be made available by the authors, without undue reservation.
