# Supplementary material for: Validity, reliability, and feasibility of the digital motor performance test (DigiMot)
Source: Front Sports Act Living. 2025 Oct 28;7:1688017. doi: 10.3389/fspor.2025.1688017 (PMC12602381; doi:10.3389/fspor.2025.1688017)
Supplement: Supplementary file 1 [file Datasheet1.docx]

Supplementary Material

## Supplementary Figures


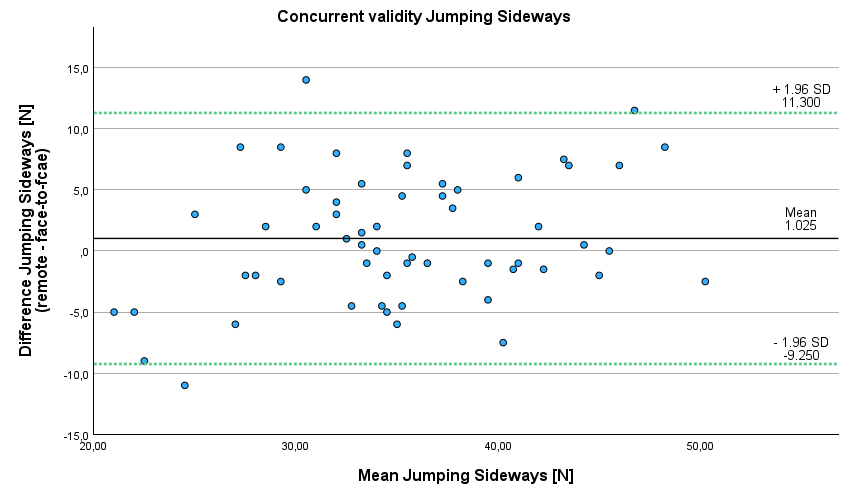


**Supplementary Figure 1.** Bland–Altman plot illustrating the concurrent validity of the Jumping Sideways. The mean number of repetitions from the remote and face-to-face assessments is shown on the x-axis, and the difference between the two assessments is shown on the y-axis. The solid black line represents the overall mean difference, while the dotted green lines indicate the upper (+1.96 SD) and lower (–1.96 SD) limits of agreement.


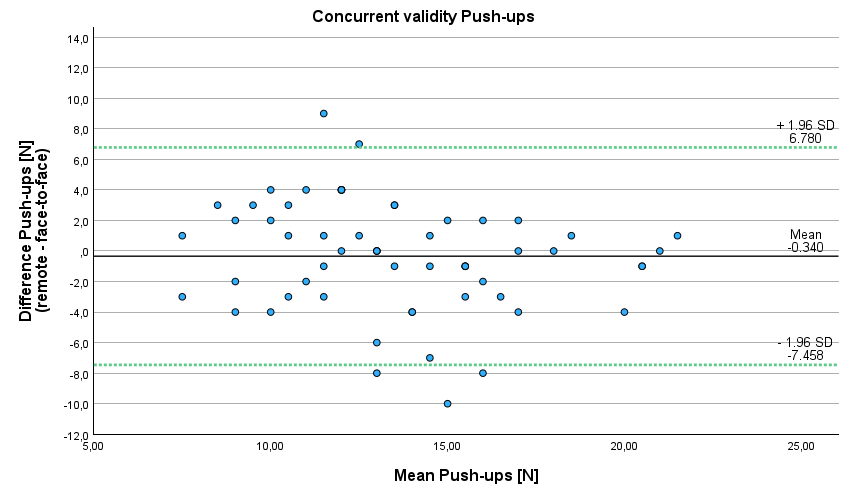


**Supplementary Figure 2.** Bland–Altman plot illustrating the concurrent validity of the Push-ups. The mean number of repetitions from the remote and face-to-face assessments is shown on the x-axis, and the difference between the two assessments is shown on the y-axis. The solid black line represents the overall mean difference, while the dotted green lines indicate the upper (+1.96 SD) and lower (–1.96 SD) limits of agreement.


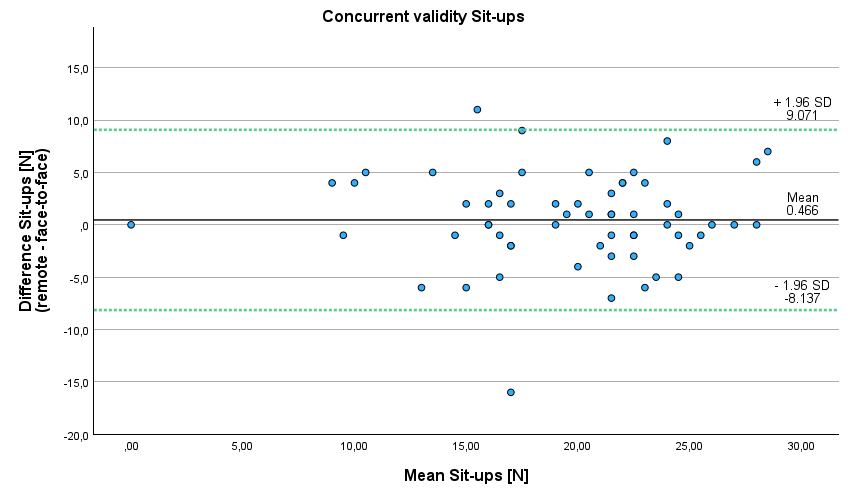


**Supplementary Figure 3.** Bland–Altman plot illustrating the concurrent validity of the Sit-ups. The mean number of repetitions from the remote and face-to-face assessments is shown on the x-axis, and the difference between the two assessments is shown on the y-axis. The solid black line represents the overall mean difference, while the dotted green lines indicate the upper (+1.96 SD) and lower (–1.96 SD) limits of agreement.


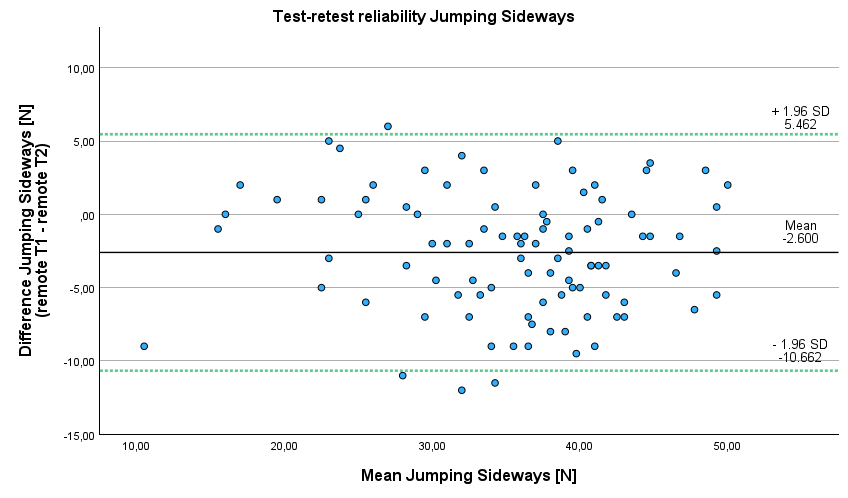


**Supplementary Figure 4.** Bland–Altman plot illustrating the test-retest reliability of the Jumping Sideways. The mean number of repetitions from the remote and face-to-face assessments is shown on the x-axis, and the difference between the two assessments is shown on the y-axis. The solid black line represents the overall mean difference, while the dotted green lines indicate the upper (+1.96 SD) and lower (–1.96 SD) limits of agreement.


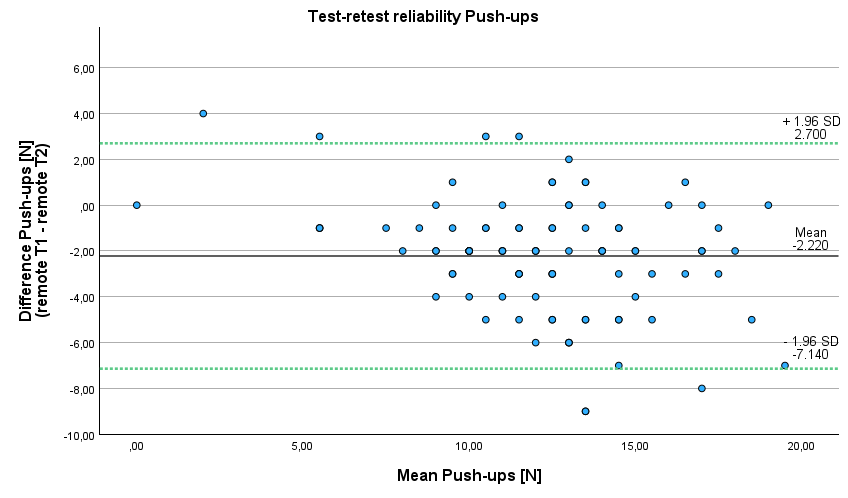


**Supplementary Figure 5.** Bland–Altman plot illustrating the test-retest reliability of the Push-ups. The mean number of repetitions from the remote and face-to-face assessments is shown on the x-axis, and the difference between the two assessments is shown on the y-axis. The solid black line represents the overall mean difference, while the dotted green lines indicate the upper (+1.96 SD) and lower (–1.96 SD) limits of agreement.


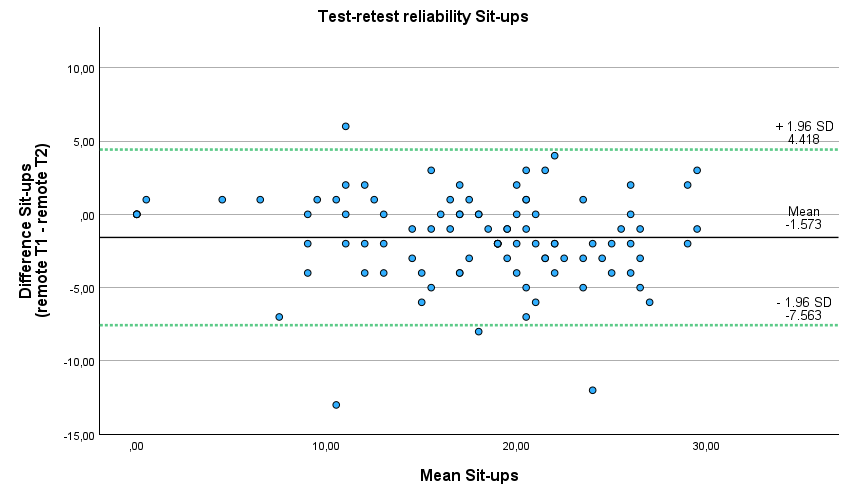


**Supplementary Figure 6.** Bland–Altman plot illustrating the test-retest reliability of the Sit-ups. The mean number of repetitions from the remote and face-to-face assessments is shown on the x-axis, and the difference between the two assessments is shown on the y-axis. The solid black line represents the overall mean difference, while the dotted green lines indicate the upper (+1.96 SD) and lower (–1.96 SD) limits of agreement.


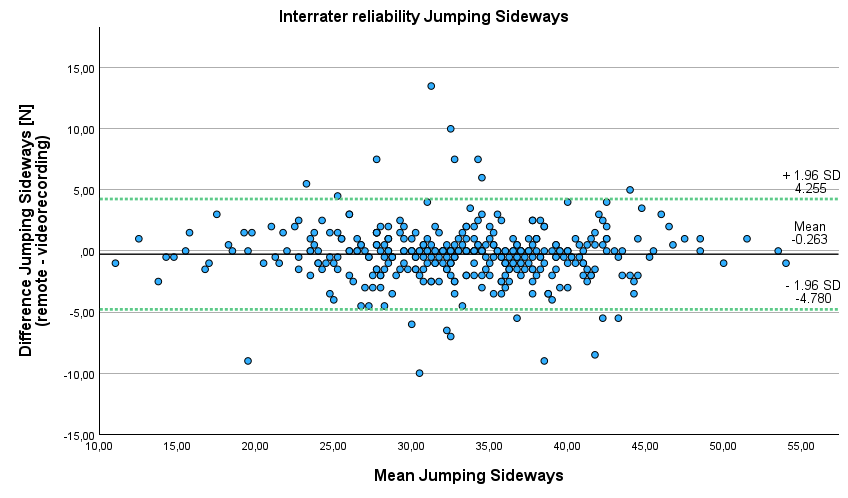


**Supplementary Figure 7.** Bland–Altman plot illustrating the interrater reliability of the Jumping Sideways. The mean number of repetitions from the remote and face-to-face assessments is shown on the x-axis, and the difference between the two assessments is shown on the y-axis. The solid black line represents the overall mean difference, while the dotted green lines indicate the upper (+1.96 SD) and lower (–1.96 SD) limits of agreement.


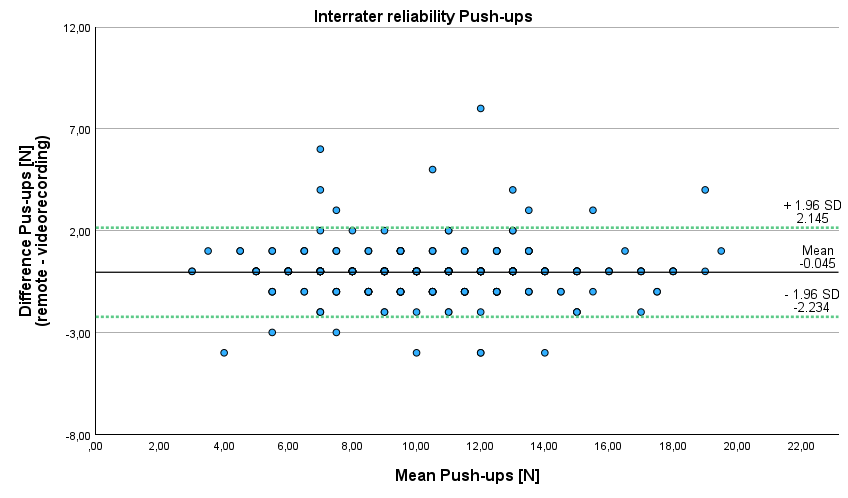


**Supplementary Figure 8.** Bland–Altman plot illustrating the interrater reliability of the Push-ups. The mean number of repetitions from the remote and face-to-face assessments is shown on the x-axis, and the difference between the two assessments is shown on the y-axis. The solid black line represents the overall mean difference, while the dotted green lines indicate the upper (+1.96 SD) and lower (–1.96 SD) limits of agreement.


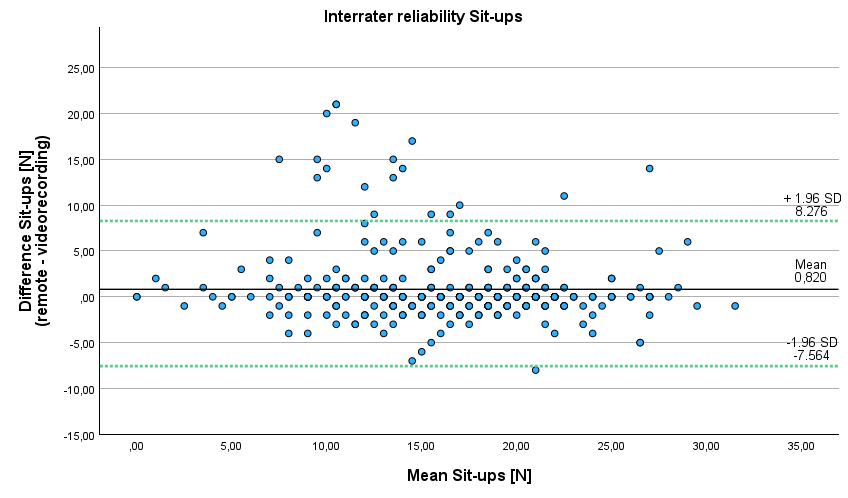


**Supplementary Figure 9.** Bland–Altman plot illustrating the interrater reliability of the Sit-ups. The mean number of repetitions from the remote and face-to-face assessments is shown on the x-axis, and the difference between the two assessments is shown on the y-axis. The solid black line represents the overall mean difference, while the dotted green lines indicate the upper (+1.96 SD) and lower (–1.96 SD) limits of agreement.
